# Impact of Tendon Gap on Decision-Making in Acute Achilles Tendon Rupture: A Systematic Review

**DOI:** 10.7759/cureus.91902

**Published:** 2025-09-09

**Authors:** Mohamed Elbeshbeshy, Mohamed Khalafallah, Adam Fell, Andrew Davies, Mohamed Hashem

**Affiliations:** 1 Trauma and Orthopaedics, Wirral University Teaching Hospital NHS Foundation Trust, Wirral, GBR; 2 Faculty of Medicine, Alexandria University, Alexandria, EGY; 3 Trauma and Orthopaedics, St Mary’s Hospital, London, GBR; 4 Trauma and Orthopaedics, Imperial College London, London, GBR; 5 Orthopaedics, Frimley Health NHS Foundation Trust, London, GBR

**Keywords:** achilles rupture, achilles tendon injury, achilles tendon rehabilitation, achilles tendon repair, conservative management of achilles tendon, tendo achilles rupture

## Abstract

Acute Achilles tendon rupture (AATR) is a prevalent injury that significantly impacts clinical decision-making, particularly concerning the initial gap size following the rupture. This review aims to evaluate the influence of initial gap size on treatment decisions in managing acute Achilles tendon complete ruptures.

A comprehensive search was conducted across four electronic databases - PubMed, Scopus, Web of Science (WoS), and the Cochrane Library - as well as Google Scholar. Two independent reviewers assessed the methodological quality of each included study using the Newcastle-Ottawa Scale (NOS), with studies classified as poor (0-3 stars), fair (4-6 stars), or good (7-9 stars). Discrepancies between reviewers were resolved by the senior author. Initially, 531 articles were identified; after removing 183 duplicates, 348 articles remained for title and abstract screening. From these, 305 were excluded, resulting in 43 studies selected for full-text assessment. Ultimately, eight studies, encompassing a total of 679 patients, met the specified inclusion and exclusion criteria and were included in the final synthesis. The Preferred Reporting Items for Systematic Reviews and Meta-Analyses (PRISMA) flow diagram illustrates the study selection process.

Eight studies, encompassing 679 patients, were included. Gap-based treatment protocols were commonly employed, with a threshold of 5-10 mm influencing surgical decision-making. Re-rupture rates were consistently low across both operative and nonoperative groups, with no statistically significant differences reported in most studies. While several studies found no correlation between gap size and the Achilles tendon Total Rupture Score (ATRS), others demonstrated that gaps >5 mm or >10 mm were associated with significantly worse functional outcomes or plantarflexion strength deficits. One study highlighted that the location of the rupture relative to the calcaneal enthesis may better predict long-term strength and fatigue scores than gap size alone. The quality of the included studies varied, with six rated as fair quality and two as good quality; none were classified as poor quality.

Larger initial gaps in AATR (especially >5-10 mm) tend to be associated with marginally poorer functional outcomes and a higher risk of re-rupture, though evidence is mixed. Many clinicians, therefore, use a conservative cut-off (<10 mm) when selecting nonoperative treatment. However, patients with large gaps may still do well with modern functional protocols. In decision-making, the tendon gap should be considered alongside patient activity level and preferences. The literature is limited by small cohorts and variable methods; future trials are needed to define optimal gap-based guidelines.

## Introduction and background

Acute Achilles tendon rupture (AATR) is a common injury, particularly among active individuals in their 30s to 50s who play sports, and it often results in lower limb disability. AATR incidence has been increasing steadily, affecting up to 40 patients per 100,000 population annually [1].

AATR presents significant challenges in clinical decision-making regarding management strategies. Treatment options include operative and non-operative approaches. Operative management consists of either open repair or minimally invasive surgery (MIS). In contrast, non-operative management involves functional rehabilitation to allow contact between tendon ends, thereby facilitating healing. This is achieved by placing the foot in a cast or orthosis in full equinus, followed by gradual dorsiflexion for two to three months [2-4].

Although operative management permits a faster return to work and is associated with lower re-rupture rates (RR 0.27 for open repair and RR 0.14 for MIS), it exhibits higher risks of wound infections, deep vein thrombosis (DVTs), and nerve injuries [5]. On the other hand, non-operative management, following an early weight-bearing protocol in selected patients, was found to yield similar functional outcomes compared to operative management [6]. In particular, functional bracing was found to have similar Achilles tendon Total Rupture Score (ATRS) and re-rupture rates to casting techniques [2].

The initial gap size following a rupture is an easily measurable factor, using dynamic ultrasound, that may influence treatment outcomes [7]. While various studies have explored the implications of gap size on healing and functional recovery, the selection criteria for operative management in many studies tended to exclude larger tendo-Achilles (TA) gap sizes, at different thresholds ranging from 5 mm to 5 cm [8,9]. Hence, the relationship between initial gap size and decision-making processes remains under-examined in the literature.

This review aims to synthesize existing literature on how the initial gap size affects clinical decisions in the management of AATRs. By analysing the impact of gap size on surgical versus conservative treatment choices, as well as on recovery protocols and rehabilitation strategies, this review seeks to clarify the role that gap size plays in shaping clinical pathways. A comprehensive understanding of this relationship is essential for optimizing treatment approaches and improving patient outcomes in this prevalent injury.

## Review

Methods

Data Sources and Search Strategy

This systematic review was conducted following the Preferred Reporting Items for Systematic Reviews and Meta-Analyses (PRISMA) guidelines [10]. We also prospectively registered our study protocol in the PROSPERO database (CRD42024584609). A comprehensive literature search was performed in September 2024 using four electronic databases - PubMed, Scopus, Web of Science (WoS), and Cochrane Library - along with Google Scholar. To minimize the likelihood of missing relevant studies, we developed a targeted search strategy specific to each database (Table 3, see Appendix).

Additionally, manual searching was undertaken through: (a) screening articles labelled as “similar articles” on PubMed for each study ultimately included in our review; (b) examining the reference lists of the final included articles; and (c) employing Google searches with the exact keywords used in the original database queries.

Eligibility Criteria and Selection Process

The inclusion criteria were: (a) clinical trials and observational studies; (b) studies published in English; and (c) studies that mentioned the gap size in acute TA rupture and its impact on the functional outcomes in nonoperatively treated patients. The exclusion criteria were: (a) case reports, case series, animal, and in vivo studies; (b) studies published in a non-English language; (c) studies that discussed chronic TA rupture only; and (d) low-quality studies. Following the previously mentioned criteria, two authors independently reviewed the titles, abstracts, and full texts of each retrieved study, with any disagreements resolved by the senior author.

Data Extraction

The following data were extracted from each study in a spreadsheet: first author, publication year, study design, number of patients treated nonoperatively, mean follow-up (months), aim of the study, outcome measures, mean ATRS, mean modified Leppilahti score (mLS), mean tendon gap, and re-rupture rates. Two reviewers conducted the data extraction, and any disagreements were resolved through discussion or by consulting the senior author.

Risk of Bias

Two reviewers independently assessed the methodological quality of each included study using the Newcastle-Ottawa Scale (NOS) [11]. Studies receiving 0-3 stars were classified as poor quality, those with 4-6 stars as fair, and those scoring 7-9 stars as good. The senior author resolved any disagreements between the reviewers.

Data Synthesis

A narrative synthesis was employed to summarize the evidence on the impact of gap distance in AATR on decision-making and functional outcomes. Extracted data were organized thematically to address the study objectives, focusing on reported functional outcomes such as validated patient-reported outcome measures, re-rupture rates, and complications. Key findings were grouped and compared based on study characteristics, including patient demographics, methodology, and gap measurement techniques. Differences and similarities across studies were highlighted to provide a comprehensive overview, and the implications of the findings were interpreted to aid clinical decision-making.

Results

Study Selection and Data Extraction

**Figure 1:**
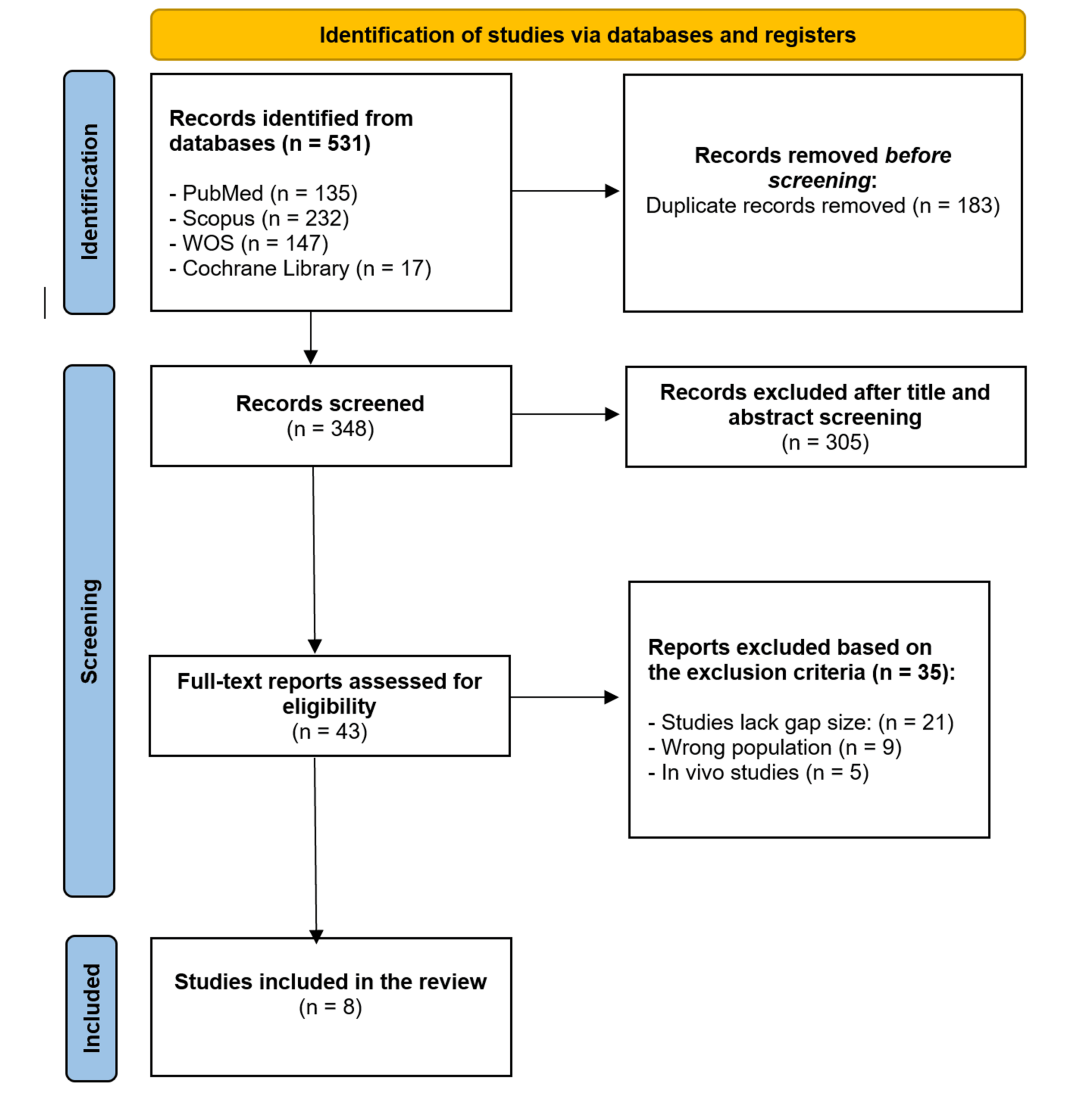
Flowchart of the study selection process

A total of 531 articles were initially identified. After removing 183 duplicate records, 348 articles remained for title and abstract screening. From these, 305 were excluded, resulting in 43 studies selected for full-text assessment. Ultimately, eight studies, encompassing a total of 679 patients, met the specified inclusion and exclusion criteria and were included in the final synthesis. Figure 1 illustrates the PRISMA flow diagram outlining the study selection process, and a summary of the included studies can be found in Table 1.

**Table 1:** Summary of included studies ATRS: Achilles tendon rupture score; TA: Tendo-Achilles; NA: Not applicable; mLS: modified Leppilahti score

Author, year (Ref)	Design	Non-operative	Operative	Aim of the study	Gap size threshold for operative management	Outcome measures	Follow-up (months)	ATRS (mean)	mLS	Tendon gap in mm (range)	Re-rupture rates
Hutchison et al. (2015) [12]	Prospective	211	62	Re-rupture rates in patients with <10 mm tendon gap treated with functional rehabilitation protocols. Operative if age <55 years old, and complete rupture with a gap >10 mm	10 mm	Re-rupture and ATRS at 4, 6, and 9 months; Cost-benefit analysis	9	72.4	-	-	1.10%
Kotnis et al. (2006) [8]	Prospective	58	67	Functional outcome following surgical and non-operative treatment with serial casting and functional rehabilitation in patients with TA <5 mm treated non operatively, >5 mm treated surgically	5 mm	Re-rupture, complications	12	-	-	-	3.40%
Lawrence et al. (2017) [13]	Prospective	38	0	Effect of tendon gap on peak torque deficit and ATRS in patients with a tendon gap of <10 mm and >10 mm at 6 months	NA	ATRS	6	87.2	-	7.1 (0-36)	-
Mubark et al. (2020) [14]	Prospective	56	0	Functional outcomes in nonoperatively managed TA rupture at 12 months with correlation between tendon rupture gap, ATRS, and peak torque deficit	NA	ATRS, Re-rupture	15.2	85.12	-	13.7	3.80%
Naskar et al. (2022) [9]	Prospective	41	0	Functional outcomes in patients with a tendon gap <50 mm treated with a functional rehabilitation protocol	50 mm	ATRS, re-ruptures, and strength assessment	12	82.1	-	-	2.43%
Qureshi et al. (2023) [15]	Retrospective	19	0	Assessment of non-operative Achilles rupture with ultrasound assessment of healing, ATRS, and mLS	NA	ATRS and mLS	73	86	71	11.4	0
Westin et al. (2016) [16]	Retrospective	24	21	Impact of gap size on re-rupture rates and functional outcomes	NA	ATRS, re-rupture	12	74.3	-	-	6.7%
Yassin et al. (2020) [17]	Prospective	82	0	Ultrasound-measured tendon gap correlated with patient-reported functional outcomes, sex, and age at 12 months in patients treated with a functional rehabilitation program	5 mm in active patients; 10 mm in others	ATRS, re-rupture	12	76	-	9 (0-43)	1.52%

Quality Assessment

The quality of the included studies varied, with six studies rated as fair quality, and two studies rated as good. None of the studies was classified as of poor quality. The most frequent source of bias was the absence of a comparison group or the lack of comparability between confounders in some studies (Table 2).

**Table 2:** Quality assessment of the included studies according to Newcastle-Ottawa Scale (NOS)

Study	Selection		Comparability	Outcomes	Total scores
Hutchison et al. (2015) [12]	3	0		3	6 - Fair
Kotnis et al. (2006) [8]	4	1		2	7 - Good
Lawrence et al. (2017) [13]	3	1		2	6 - Fair
Mubark et al. (2020) [14]	3	0		3	6 - Fair
Naskar et al. (2022) [9]	3	1		3	7 - Good
Qureshi et al. (2023) [15]	3	0		3	6 - Fair
Westin et al. (2016) [16]	3	1		2	6 - Fair
Yassin et al. (2020) [17]	3	1		2	6 - Fair

Narrative Synthesis

The included studies collectively examined the role of initial tendon gap size, measured by dynamic ultrasound, in guiding treatment decisions and predicting outcomes in AATR. While several studies support using gap size thresholds to inform operative versus non-operative strategies, others question their independent predictive value. Below, we synthesize our findings thematically.

Gap-based protocols for surgical decision-making: Several studies employed predefined gap thresholds to allocate patients to either operative or non-operative pathways.

Kotnis et al. [8] used a 5 mm cutoff, assigning patients with larger gaps to surgery (n = 67) and others to functional non-operative rehabilitation (n = 58). Both groups underwent the same postoperative protocol. At one-year follow-up, re-rupture rates were low and not statistically different between the surgical (1.5%) and nonsurgical (3.4%) groups (p = 0.60). While complications were more frequent in the operative cohort, none reached statistical significance. The authors concluded that ultrasound-guided, gap-based stratification is a safe approach that may help minimize re-ruptures.

Hutchison et al. [12] applied a more conservative approach by following the Swansea Morriston Achilles Rupture Treatment (SMART) protocol, offering surgery only to patients under 55 years with complete rupture and a gap >10 mm. Others were managed nonoperatively with functional rehabilitation. ATRS scores were nearly identical at nine months between groups (72.4 vs. 72.3), and the overall re-rupture rate was only 1.1%. The study demonstrated that patients with gaps <10 mm could achieve comparable outcomes to those treated surgically, supporting a selective surgical approach. However, the study was limited by low long-term follow-up rates, as patients were discharged upon functional recovery.

Gap size and functional outcomes in non-operative cohorts: Several studies focused on non-operative management exclusively, aiming to understand how tendon gap size affects recovery.

Lawrence et al. [13] examined 38 patients and found that those with tendon gaps >10 mm had significantly greater plantarflexion torque deficits (23.3% vs. 14.3%, p = 0.023), indicating biomechanical disadvantage. However, this did not translate into a significant difference in ATRS scores (87.2 vs. 87.4, p = 0.467), suggesting that objective strength impairments may not be reflected in short-term patient-reported function. Similarly, Mubark et al. [14] observed no significant correlation between tendon gap size and ATRS at 12-month follow-up (r = 0.091, p = 0.167), reinforcing the notion that functional recovery can occur across a wide range of gap sizes when modern rehabilitation is applied.

In contrast, Yassin et al. [17] reported a clear inverse relationship between gap size and functional outcomes. In their cohort of 82 patients, ATRS scores were significantly lower in patients with gaps >5 mm (73 vs. 82, p = 0.031) and >10 mm (70 vs. 80, p = 0.034). They also noted that older age and female sex were associated with poorer outcomes. The findings led the authors to recommend using >5 mm as a surgical threshold in active patients and >10 mm in others.

Naskar et al. [9] described the outcomes of 41 patients with gaps <50 mm treated nonoperatively using a structured Achilles rehabilitation protocol. Despite their high activity level (77% engaged in sports), patients achieved a mean ATRS of 82.1 and a low re-rupture rate (2.4%), comparable to existing literature. Heel raise performance was reduced compared to the contralateral limb (p < 0.0001), reflecting some strength deficit. However, the study lacked a surgical comparator and did not stratify outcomes by specific gap sizes, limiting interpretation.

Alternative predictors - rupture location vs. gap size: While most studies emphasized gap size, Qureshi et al. [15] highlighted rupture location relative to the calcaneal enthesis as a potentially stronger predictor of long-term outcomes. In a small cohort (n = 19) with six-year follow-up, they found no significant differences in ATRS or mLS between those with gaps <10 mm and >10 mm. However, patients with rupture sites <5 cm from the enthesis showed worse strength and fatigue scores (p < 0.05). This finding suggests that anatomical location may carry more prognostic weight than absolute gap size.

Summary: Across studies, tendon gap size appears to play a role in early clinical decision-making, especially when using defined thresholds (e.g., 5 mm or 10 mm) to triage patients for surgery. However, its value as a standalone predictor of long-term function is inconsistent. Objective deficits in strength may occur with larger gaps, but patient-reported outcomes are often preserved with appropriate rehabilitation. Additionally, rupture location and patient-specific factors (e.g., age, sex, activity level) are reported to influence recovery and should be considered alongside gap size when guiding treatment.

Discussion

This systematic review evaluated the impact of initial Achilles tendon gap size on treatment decisions and functional outcomes in acute tendon rupture. Across eight studies involving 679 patients, several findings emerged: (a) tendon gap thresholds (commonly 5-10 mm) were frequently used to stratify patients for operative versus non-operative care; (b) re-rupture rates were consistently low in both groups; and (c) while some studies reported associations between larger gaps and diminished functional outcomes, others found no significant correlation. These findings highlight both the potential value and the limitations of using tendon gap size as a decision-making tool.

Comparison With Prior Evidence

Our review supports previous work suggesting that ultrasound-measured tendon gap can guide early management. For instance, Kotnis et al. [8] and Hutchison et al. [12] implemented gap-based protocols with thresholds of 5 mm and 10 mm, respectively, and achieved low re-rupture rates across both surgical and non-operative cohorts. These outcomes are consistent with the findings of Westin et al. [16], who also observed worse ATRS and higher re-rupture rates in patients with gaps >5 mm and >10 mm when managed nonoperatively.

However, other studies, such as those by Lawrence et al. [13], Mubark et al. [14], and Qureshi et al. [15], reported no statistically significant relationship between gap size and functional outcome scores (e.g., ATRS or mLS), even for gaps exceeding 10 mm. This divergence likely reflects variations in methodology, patient demographics, rehabilitation strategies, and the multifactorial nature of TA ruptures, supporting the findings of Xergia et al. [18]. 

Explaining Heterogeneity

Heterogeneity across studies is a significant consideration. Key sources of variability include (a) measurement technique: some studies assessed gap size with the ankle in neutral position, while others used the plantarflexed position. As shown by Qureshi et al. [15], gap measurements can vary significantly with ankle and knee positioning - from 12 mm in neutral ankle position, to 5 mm in maximal plantar flexion, and down to 2 mm in maximum equinus with 90 degrees of knee flexion; (b) patient populations: outcomes may differ by age, sex, BMI, and activity level. For example, Yassin et al. [17] found that both age and sex influenced ATRS scores independent of gap size; (c) outcome measures: most studies used patient-reported outcomes (ATRS), while others included biomechanical data (e.g., torque testing), which may better reflect subtle functional deficits; (d) rehabilitation protocols: protocols varied in terms of weight-bearing timing, orthosis type, and follow-up, which may influence healing regardless of gap size.

These inconsistencies limit direct comparability and caution against using the tendon gap as a sole predictor of outcomes.

Clinical Implications

Despite mixed evidence, many clinicians continue to use tendon gap thresholds - most commonly 5-10 mm - as part of their selection criteria for operative management. This practice is pragmatic, particularly when aiming to reduce re-rupture risk or address concerns about tendon apposition. However, our review suggests that patients with larger gaps may still achieve favourable outcomes under modern, structured non-operative protocols, such as those incorporating early functional rehabilitation and weight-bearing.

Given this, the tendon gap size should be interpreted as one factor among several. Clinical decision-making should also incorporate patient goals, activity demands, rupture location, and comorbidities. Furthermore, the findings by Qureshi et al. [15] raise an important consideration: rupture proximity to the calcaneal enthesis may be a more predictive factor of long-term function than absolute gap size.

Strengths and limitations

This review was strengthened by adherence to PRISMA guidelines, prospective registration in PROSPERO, and use of validated quality assessment tools (NOS). It also provides a focused synthesis of a clinically relevant but underexplored topic. However, several limitations should be acknowledged: (a) most included studies were observational, with small sample sizes and no randomization; (b) many lacked standardization in gap measurement, blinding, or control for confounders; and (c) heterogeneity in rehabilitation protocols and outcome measures limited the meta-analysis.

Thus, while the evidence suggests trends, definitive conclusions are constrained by methodological inconsistency and potential bias.

Recommendations for future research

Future studies should aim to (a) standardize ultrasound gap measurement protocols (e.g., defined limb position and timing post-injury); (b) conduct high-quality prospective trials stratified by gap size; (c) incorporate both subjective (ATRS) and objective (strength, return-to-sport) outcomes; and (d) evaluate long-term functional and structural healing, including the role of rupture location.

Such studies would help clarify whether and how the tendon gap should influence modern treatment algorithms.

## Conclusions

Current evidence suggests that initial Achilles tendon gap size is one of several measurable factors influencing treatment selection. Some surgeons use a gap threshold (commonly 5-10 mm) to guide surgical management, citing studies that report relatively worse function and higher re-rupture rates with non-operative treatment. Conversely, other studies have shown that carefully selected patients with gaps up to 50 mm can achieve good outcomes under early functional rehabilitation protocols, challenging the predictive value of gap size alone.

Given these conflicting findings, treatment decisions should consider gap size as part of a more comprehensive assessment that also incorporates the patient’s activity level, functional demands, and individual preferences. To clarify the role of gap size in guiding management, high-quality randomized controlled trials are needed to rigorously evaluate gap-based treatment criteria.

## Appendices

**Table 3:** Search strategies

Database	Search strategy	Records retrieved
PubMed	(("achilles tendon"[MeSH Terms] OR ("achilles"[All Fields] AND "tendon"[All Fields]) OR "achilles tendon"[All Fields]) AND ("injuries"[MeSH Subheading] OR "injuries"[All Fields] OR "trauma"[All Fields] OR "wounds and injuries"[MeSH Terms] OR ("wounds"[All Fields] AND "injuries"[All Fields]) OR "wounds and injuries"[All Fields] OR "trauma s"[All Fields] OR "traumas"[All Fields] OR ("ruptur"[All Fields] OR "rupture"[MeSH Terms] OR "rupture"[All Fields] OR "ruptured"[All Fields] OR "ruptures"[All Fields] OR "rupturing"[All Fields]) OR ("acute"[All Fields] OR "acutely"[All Fields] OR "acutes"[All Fields]) OR (("traumatic"[All Fields] OR "traumatically"[All Fields] OR "traumatism"[All Fields] OR "traumatisms"[All Fields] OR "traumatization"[All Fields] OR "traumatizations"[All Fields] OR "traumatize"[All Fields] OR "traumatized"[All Fields] OR "traumatizes"[All Fields] OR "traumatizing"[All Fields]) AND ("acute"[All Fields] OR "acutely"[All Fields] OR "acutes"[All Fields]) AND ("ruptur"[All Fields] OR "rupture"[MeSH Terms] OR "rupture"[All Fields] OR "ruptured"[All Fields] OR "ruptures"[All Fields] OR "rupturing"[All Fields])) OR ("injurie"[All Fields] OR "injuried"[All Fields] OR "injuries"[MeSH Subheading] OR "injuries"[All Fields] OR "wounds and injuries"[MeSH Terms] OR ("wounds"[All Fields] AND "injuries"[All Fields]) OR "wounds and injuries"[All Fields] OR "injurious"[All Fields] OR "injury s"[All Fields] OR "injuryed"[All Fields] OR "injurys"[All Fields] OR "injury"[All Fields])) AND (("gap"[All Fields] AND "size"[All Fields]) OR (("tendinopathy"[MeSH Terms] OR "tendinopathy"[All Fields] OR "tendonitis"[All Fields] OR "tendon s"[All Fields] OR "tendonous"[All Fields] OR "tendons"[MeSH Terms] OR "tendons"[All Fields] OR "tendon"[All Fields]) AND "gap"[All Fields]))) AND (y_10[Filter])	135
Scopus	TITLE-ABS-KEY ((achilles AND tendon) AND (trauma OR rupture OR acute OR (traumatic AND acute AND rupture) OR injury) AND (gap AND size OR tendon AND gap)) AND (LIMIT-TO (SUBJAREA, "MEDI")) AND (LIMIT-TO (LANGUAGE, "English"))	232
WOS	(Achilles tendon) AND (trauma OR rupture OR acute OR (traumatic acute rupture) OR injury) AND (gap size OR tendon gap) (All Fields)	147
Cochrane CENTRAL	((Achilles tendon) AND (trauma OR rupture OR acute OR (traumatic acute rupture) OR injury) AND (gap size OR tendon gap)):ti,ab,kw	17
Total= 531; Removed duplicates= 183; Title and abstract screening= 348; Full text screening= 43; Final included= 8		
